# Aplicaciones de la tomografía computarizada de haz cónico de la estructura craneofacial en especialidades médicas. Una revisión

**DOI:** 10.21142/2523-2754-1001-2022-100

**Published:** 2022-03-30

**Authors:** Agustín Muñoz-Galván, Gustavo Adolfo Fiori-Chíncaro, Ana María Agudelo-Botero

**Affiliations:** 1 Division de Radiologia Bucal y Maxilofacial, Universidad Cientifica del Sur. Lima, Peru. agustinmunozg@yahoo.com Universidad Científica del Sur Division de Radiologia Bucal y Maxilofacial Universidad Cientifica del Sur Lima Peru agustinmunozg@yahoo.com; 2 Instituto Latinoamericano de Altos Estudios en Estomatologia (ILAE). Lima, Peru. gfiori@ilaeperu.com, ortoamariabotero@gmail.com Instituto Latinoamericano de Altos Estudios en Estomatologia (ILAE) Lima Peru gfiori@ilaeperu.com ortoamariabotero@gmail.com

**Keywords:** tomografía computarizada de haz Cónico, tomografía computarizada cone beam, maxilofacial, especialidades médicas, odontología, tomografía computarizada, cone-beam computed tomography, cone beam CT, maxillofacial, medical specialties, dentistry, dental, multislice computed tomography, multidetector computed tomography

## Abstract

La tomografía computarizada de haz cónico (TCHC) de la estructura craneofacial ha demostrado su utilidad en el área odontológica durante las últimas décadas, por lo que se ha convertido en una herramienta fundamental para el diagnóstico, la planeación y la evaluación del tratamiento. Aunque no fue desarrollada para uso exclusivo en odontología como comúnmente se considera, es utilizada en otras áreas como la medicina. Específicamente, se emplea en el área de cabeza y el cuello, donde interactúan diferentes especialidades médicas, y a la vez estas interactúan con especialidades odontológicas; por tanto, conocer las ventajas de la TCHC sobre diferentes tecnologías imagenológicas en el área médica es necesario. El propósito de esta revisión fue describir las aplicaciones de observación, diagnóstico, planeación y evolución de tratamientos mediante la TCHC maxilofacial en diferentes especialidades médicas. Este trabajo destaca el uso de la TCHC en las diferentes aplicaciones médicas y resalta dónde radica su mayor utilidad en comparación con otras tecnologías.

## Abstract (en)

Cone-beam computed tomography (CBCT) of the craniofacial structure has demonstrated its usefulness in the dental area during the last decades, thus it has become a fundamental tool in the diagnosis, planning, and evaluation of treatment, and although it was not developed for the exclusive use in dentistry, as it is commonly considered, it is used in other areas such as medicine. It is specifically used in the head and neck area where different medical specialties interact, and at the same time these interact with dental specialties, so knowing the advantages of CBCT over different imaging technologies in the medical area is necessary. The purpose of this review was to describe the applications of observation, diagnosis, planning, and evolution of treatments using maxillofacial HSCT in different medical specialties. This work highlights the use of TCHC in different medical applications and highlights where it is most useful compared to other technologies.

## INTRODUCCIÓN

La tomografía computarizada de haz cónico (TCHC) es una herramienta radiográfica que durante aproximadamente 20 años ha demostrado su utilidad en el área de la odontología ^1^^,^^2^. Se tiene la percepción que la tecnología TCHC fue desarrollada para la imagenologia maxilofacial y dental exclusivamente, sin embargo inicialmente se enfocaba en aplicaciones para angiología en el área médica, en la cual la resolución del tejido blando no era de relevancia, pero sí la alta resolución espacial que proporciona ^1^^,^^2^. 

La TCHC provee un método alternativo a la tomografía multisensor o tomografía médica, sobre todo en la revisión de patologías maxilofaciales, incluidos el cuello, la región paranasal, la extracraneal y la temporal ^2^^,^^3^. En trauma maxilofacial, la TCHC ofrece una mayor precisión y sensibilidad para valorar las regiones maxilar y mandibular ^3^^,^^4^. 

Se han desarrollaron equipos de TCHC para procedimientos intervencionistas; angiografía; guía de radioterapia; procedimientos en columna, región abdominal y toráxica; y procedimientos ortopédicos, esto con equipos con un brazo en C ^5^^,^^6^. También se están valorando aplicaciones para mamografías, neurorradiología, radiología intervencionista, entre otras ^7^^,^^8^. Así mismo, son de utilidad para la evaluación pre y posoperatoria en traumatología y neurocirugía ^7^^-^^9^. 

El avance en las tecnologías imagenológicas incrementa la habilidad y la posibilidad de identificar hallazgos incidentales. La incapacidad de identificar, dar seguimiento, informar y favorecer el tratamiento, según el caso, se refleja de manera negativa en el paciente y puede tener implicaciones legales ^10^^-^^12^. Al aumentar el uso por frecuencia y las indicaciones de la TCHC, Academia Americana de Radiología Oral y Maxilofacial (AAOMR) y la Academia Europea de Radiología Dentomaxilofacial (EADMFR) han redactado lineamientos en los que comunican que, además del conocimiento del área maxilofacial, también es necesario el de las estructuras adyacentes ^13^^,^^14^. En los lineamientos de la AAOMR se menciona que el volumen obtenido se debe evaluar a su mayor potencial y que, a partir de campos de visión medianos (FOV, por siglas en inglés: *field of view*), debe incluir regiones de cabeza y cuello que pueden no estar relacionadas con el área odontológica, como senos paranasales, oído medio, columna cervical, fosas craneales, y para lo cual debe participar un especialista en radiología oral y maxilofacial (RMF) ^12^^,^^13^.

Es necesario enfatizar la importancia de los hallazgos incidentales para el especialista en RMF con relación a las condiciones médicas que pueden requerir la referencia de un especialista medico, y en algunos casos incluso atención inmediata ^11^^,^^12^. Estudios anteriores han demostrado el aumento de reportes de hallazgos incidentales al explorar volúmenes de TCHC maxilofacial ^11^^,^^12^^,^^15^.

Por lo expuesto, el propósito de esta revisión fue describir las aplicaciones de observación, diagnóstico, planeación y evolución de tratamientos con la TCHC maxilofacial en diferentes especialidades médicas. Este trabajo destaca estos usos de la TCHC y resalta donde está su mayor utilidad en comparación con otras tecnologías.

## MATERIALES Y MÉTODOS

Se realizó una búsqueda de la literatura en las principales fuentes de información, como Medline (vía PubMed), Elsevier (vía Science Direct) y SciELO, usando los términos de búsqueda “tomografía computarizada de haz cónico”, “tomografía computarizada cone beam”, “maxilofacial” y “especialidades médicas”, con una limitación de fecha de los últimos 10 años hasta enero del 2020. Los textos seleccionados debían incluir información referente a aplicaciones de TCHC de la estructura maxilofacial en especialidades médicas. De los 370 artículos obtenidos luego de la búsqueda, se valoró cuáles aportaban información tomográfica de la estructura maxilofacial que se aplique en especialidades médicas de manera intencionada, novedosa y donde pudo considerarse como primera elección imagenológica. En total, se seleccionaron 74 artículos para referencias.

### Aplicación por especialidades médicas en las que la TCHC presenta ventajas sobre la tomografía computarizada multicorte TCM

El interés por utilizar la TCHC para uso diagnóstico, intraoperatorio y posoperatorio va en aumento en regiones fuera de lo maxilofacial. En la región de la cabeza y el cuello, que tienen una estructura ósea compleja, poder distinguir de manera precisa el detalle anatómico la convierte en una herramienta de gran utilidad ^12^^,^^16^. En diferentes especialidades médicas se utiliza la TCHC maxilofacial con fines médicos para el propósito de diagnóstico, observación, diagnóstico diferencial, planeación y evolución de tratamiento, al ser de mayor utilidad que la TCM debido a sus ventajas ^2^^,^^16^. 

Diferentes estudios han comparado la calidad de imagen de la TCHC con la TCM, a través de diferentes variables como la función de transferencia de modulación, que valora la nitidez y la resolución espacial según la capacidad de contraste entre estructuras contiguas en la imagen producida por el equipo. Se trata de evaluaciones objetivas y cuantitativas realizadas por cálculos matemáticos al diferenciar los pares de líneas (lp/cm) que puede distinguir claramente el equipo, y en las que la TCHC se mostró superior a la TCM, por su mayor resolución espacial en el detalle fino de las estructuras anatómicas ^17^^-^^19^. 

Esta resolución también ha permitido que se observen hallazgos incidentales fuera del área maxilofacial que son de importancia en diferentes grados de atención, desde referir, monitorear o dar seguimiento, hasta solo mencionar en el reporte ^11^^,^^12^^,^^20^. Entre las características que han favorecido que se adopte la TCHC maxilofacial están, principalmente, la baja dosis de radiación efectiva, la limitación de artefactos metálicos y la alta resolución espacial ^17^^,^^18^^,^^21^^,^^22^. 

Algunos autores relacionan el uso en medicina de radiación ionizante de baja dosis con el aumento en el riesgo de cáncer, que puede contribuir hasta en un 1,5% a 2,0% de los casos, un mayor riesgo de presentar tumores de sistema nervioso central (SNC) y, en población pediátrica, una incidencia en general de cáncer -incluidos los hematológicos, linfoproliferativos y tumores malignos de SNC- de 1,5 veces mayor a la esperada y, para todos los tumores de cerebro, un incremento relativo de riesgo de dos a cuatro veces en los pacientes que han recibido mayores dosis. La incidencia de cáncer en SNC permanece elevada durante 10 o más años tras la toma de la tomografía computarizada (TC) ^23^^-^^27^.

El principio de Alara (por sus siglas en inglés: *as low as reasonably achievable*) tan bajo como sea razonablemente alcanzable debe procurarse, y deben considerase las medidas de exposición a radiación ionizante. La dosis efectiva es la medida que la Comisión Internacional de Protección Radiológica (ICRP, por sus siglas en inglés) recomienda para visualizar los riegos a diferentes proyecciones de radiación ionizante ^21^^,^^28^. La TCHC, hasta el año 2015, no se relacionó directamente con la posibilidad de causar cáncer ^26^. 

La TCHC presenta como una de sus grandes ventajas sobre la TCM el obtener información de la anatomía en 3D con la menor dosis de exposición a radiación ionizante ^26^^,^^29^. Un estudio de la región maxilofacial con TCM médica puede producir una dosis efectiva de entre 474 y 1160 mSv, en comparación con la TCHC, que lo puede hacer con una menor dosis efectiva (13 a 82 mSv por estudio) ^28^^,^^29^. Es importante mencionar que no existen a la fecha estudios prospectivos que relacionen exposiciones menores a 100 mSv con el riesgo de cáncer, y la TCHC tiene dosis muy inferiores a este parámetro ^26^^,^^28^.

En diferentes regiones de la estructura craneofacial, se pueden utilizar protocolos de baja radiación de la TCHC con utilidad médica que proporcionan una imagen de calidad y de utilidad diagnóstica ^30^^-^^32^. Nardi *et al*. ^22^ realizaron una revisión sistemática de estudios en la literatura hasta el 12 de noviembre del 2017 y evaluaron la dosis de radiación en estudios con TCHC en regiones no dentales. Como resultado, encontraron una dosis efectiva promedio para estudio de oído de 178.2 mSv; para senos paranasales, de 119.0 mSv; y para columna cervical, de 248 mSv.

Por lo expuesto hasta el momento, la TCHC puede utilizarse en diferentes especialidades médicas, como la oftalmología, en la cual los cirujanos evalúan diferentes estructuras anatómicas antes de intervenciones quirúr-gicas para evitar complicaciones u ocasionar lesiones, como en el caso del trayecto del nervio infraorbitario y los vasos infraorbitarios desde su origen en la fosa craneal anterior hasta su salida en el foramen infraorbitario y su distribución en cara, que es un área que requiere procedimientos de anestesia en esta especialidad, en cirugía plástica y cirugía maxilofacial ^33^. Es importante en procedimientos para descomprimir al nervio óptico donde su relación con la arteria carótida interna y las estructuras óseas circundantes son de importancia para valorar los abordajes quirúrgicos transesfenoidal y transetmoidal para su descomprensión, en la que la TCHC proporciona una resolución submilimétrica con una dosis efectiva menor ^34^.

En oftalmología, la epífora es de las consultas más frecuentes y una de sus causas puede ser la obstrucción o drenaje insuficiente del conducto nasolagrimal. Para un diagnóstico correcto y la evaluación preoperatoria, comúnmente, se utilizan estudios 2D con medio de contraste llamados dacriocistografía, pero estos solo aportan información del conducto y no de las estructuras anatómicas relacionadas, donde se ha observado que algunas patologías de la cavidad nasal o los senos paranasales pueden causar la obstrucción. La TCHC es de utilidad para explorar la anatomía de estas áreas ^35^^,^^36^.

La osteodontoqueratopróstesis es un procedimiento de dos etapas en el que el oftalmólogo y el cirujano maxilofacial trabajan en conjunto para tratar la ceguera severa cuando no hay alternativas de tratamiento, al adaptar un cilindro en una pieza dental y fijarlo en la órbita. Se utiliza la TCHC para realizar una evaluación preoperatoria del sitio de donación, que son caninos y premolares superiores e inferiores, incluyendo en bloque la pieza dental y el hueso alveolar, así como la evaluación de la órbita. También se realiza una TCHC posoperatoria en diferentes ocasiones para dar seguimiento a la estabilidad del implante y valorar cualquier reabsorción del complejo hueso-lámina dura-diente, que al ser excesiva puede causar hipotonía, extrusión del cilindro óptico, endoftalmitis e inflamación crónica. Esta evaluación debe realizarse durante varios años, por lo que la TCHC se puede realizar a un nivel submilimétrico con una menor dosis efectiva ^37^^,^^38^.

En oncología, la TCHC muestra utilidad en el estudio del cáncer oral donde la resonancia magnética (IRM) o la TCM predominan en su estudio y la tomografía de emisión de positrón (PET), junto con TCM o con TCHC, muestran buenos resultados para su estudio y mejor estadificación del cáncer. En diferentes estudios que destacan las ventajas y desventajas de las diferentes tecnologías, se encuentra que la TCHC muestra mayor sensibilidad comparable a SPECT y mejor valor predictivo negativo a la invasión del hueso cortical por el cáncer; también se reporta que la TCHC muestra una precisión comparable a IRM/TCM y gammagrafía ósea. Qiao *et al.*^39^ realizaron una revisión sistemática y un metaanálisis para evaluar las diferentes tecnologías imagenológicas en la precisión para diagnosticar la invasión mandibular por cánceres de cabeza y cuello, y llegaron a la conclusión de que el SPECT era la mejor opción para excluir invasión mandibular debido a su mayor sensibilidad. Además, para la confirmación, señalaron a la TCM y la IRM como apropiadas, y que la TCHC y la SPECT tienen mayor capacidad diagnóstica preoperatoria de invasión mandibular que otras tecnologías, con un ventaja considerable de la TCHC sobre la SPECT ^39^^-^^42^.

### TCHC de la estructura craneofacial en otorrinolaringología

La TCHC mostró dosis efectivas menores, con una mejor calidad de imagen en comparación con protocolos de baja radiación de la TCM en maxilares; en oído, con solo la mitad de la dosis efectiva; y en cráneo y columna cervical, con solo el diez por ciento de la dosis efectiva ^(29, 30, 43)^. Las características que favorecen la utilidad de la TCHC en otorrinolaringología son una dosis efectiva menor a la TCM; la resolución espacial, que es mayor que la de la TCM; su menor afección, debido a artefactos metálicos que interfieran con la calidad de imagen que es ventajoso para evaluaciones posoperatorias de colocación de prótesis o material quirúrgico, y su alta definición ósea ^28^^,^^44^.

En senos paranasales y fosas nasales, la TCHC es de utilidad para observar diferentes patologías inflamatorias o infecciosas de diferente etiología. Asimismo, muestra ventaja sobre la TCM en el estudio de la sinusitis y descartar o confirmar si es de origen odontológico, ya que la exploración de la estructura dentomaxilar es superior ^44^^,^^45^. La TCHC ha incrementado su aceptación como herramienta diagnóstica para sinusitis crónica y puede considerarse como una alternativa a la endoscopia sinusal, por ser menos invasiva, costosa y no requerir anestesia local o general en algunos casos. También se ha encontrado una correlación de los hallazgos por endoscopia sinusal y los encontrados en un estudio de TCHC de los senos paranasales para diagnosticar sinusitis crónica ^45^^,^^46^.

La TCHC proporciona imagenología puntualmente útil para valorar la posición, forma y variaciones de los senos paranasales ^47^^-^^49^. En los senos paranasales, resulta de gran importancia reconocer sus variantes anatómicas, ya que pueden influir en procesos inflamatorios u obstructivos, además de dificultar procedimientos clínicos, en especial, la cirugía endoscópica funcional. Además, pueden influir en el resultado y la seguridad del paciente en procedimientos quirúrgicos realizados en dicha área ^47^^,^^50^.

La TCHC es de utilidad en el área temporal y la evaluación de la cadena ósea del oído medio, donde las estructuras de relevancia clínica presentan medidas milimétricas y submilimétricas que no son bien observadas mediante la TCM o la IRM. Hasta el año 2018, la TCHC presentaba una resolución espacial técnica de 75 µm y, ya en el ejercicio de 150 µm, contra la resolución de 500-600 µm de la TCM, lo que permite obtener mejores imágenes de la cadena ósea para diagnosticar la mayoría de las malformaciones, lesiones traumáticas, displasias, erosión de la delgada pared laberíntica. También permite evaluar la progresión de la pérdida auditiva progresiva, la otospongiosis, la otitis crónica, el colesteatoma, entre otras patologías, para los que presenta menores falsos positivos que la TCM ^2^^,^^43^^,^^51^^-^^53^. La cuerda del tímpano porción del nervio facial dentro del hueso temporal es más fácilmente identificada por la TCHC, pues presenta una mayor sensibilidad y certeza que la TCM en su delineación, lo cual es importante ya que se trata de una vía de acceso quirúrgico común para la colocación de implantes cocleares ^54^^,^^55^.

En trastornos de respiración, se valora la vía aérea superior, ya que es de importancia en medicina por ser una estructura anatómica compleja que, al presentar anomalías funcionales o morfológicas, puede influir en patologías como la apnea obstructiva del sueño (AOS), que requiere de evaluación multidisciplinaria al intervenir el otorrinolaringólogo, el cirujano plástico, el ortodoncista y el neurólogo. Diferentes estudios han evaluado la fidelidad de la TCHC craneofacial para evaluar la vía aérea superior, como Zimmerman *et al*. ^56^, quienes realizaron una revisión sistemática hasta junio del 2015 de este tipo de estudios y hallaron una fidelidad intra e interexaminador de moderada a excelente para el cálculo del volumen en mayor grado y medición del área mínima horizontal, en la que no solo se debe medir la vía aérea superior en total, sino cada sección que incluye nasofaringe, orofaringe e hipofaringe, y para lo cual se deben definir y estandarizar los limites anatómicos de cada sección ^56^^-^^58^.

### TCHC de la estructura craneofacial en neurología y neurocirugía

La TCHC permite la evaluación submilimétrica de las estructuras óseas con una alta resolución espacial, en línea con estudios realizados por TCM, que permiten observar el volumen de estructuras óseas en la región maxilofacial, lo que ha despertado el interés por su aplicación fuera de esta área ^2^^,^^21^^,^^59^. La alta resolución espacial isotrópica de la TCHC la convierte en una herramienta diagnóstica de importancia para distinguir la compleja anatomía ósea en cráneo y cuello ^16^^,^^43^. 

Estas características son de utilidad en el área de la neurología y la neurocirugía, en las que se han evaluado diferentes aplicaciones, como dar seguimiento a la osteointegración de una fusión intersomática cervical anterior, que por lo general se realiza con radiografías 2D y TCM. Sin embargo, se evalúa la TCHC por presentar un menor grado de interferencia debido al artefacto metálico de la fijación y una menor dosis efectiva ^21^^,^^22^^,^^32^^,^^60^. Existe controversia respecto de la evaluación de hueso por medio de la TCHC, que utiliza una escala de grises en comparación con las unidades Hounsfield de la TCM. La escala de grises no permite una conversión directa a unidades Hounsfield, pero sí establecer una relación lineal entre ambas para la evaluación de las diferentes densidades de los tejidos en una TCHC, así como para mediciones que evalúan la formación de hueso ^61^^-^^63^.

La TCHC también se ha utilizado en la evaluación de la apófisis crista galli en cuanto a su tamaño y, si se encuentra neumatizada, por su importancia en procesos de otorrinolaringología o neurocirugía, ya que tiene relevancia al presentar cuadros de sinusitis, procesos inflamatorios u obstruir una vía de acceso quirúrgico para algunos tumores de la fosa craneal anterior ^64^.

La base de cráneo y su complicada anatomía son importantes para estas especialidades por su relevancia clínica y radiológica, y la TCHC resulta de utilidad. Adicionalmente a la anatomía normal, diferentes agujeros y conductos poco frecuentes han sido mencionados en la literatura; por ejemplo, foramen meningo orbital, *canaliculus innominatus*, canal cráneofaringeo, entre otros que son de importancia clínica al evitar diagnósticos equivocados y tomarlos en cuenta en los procesos quirúrgicos ^65^^-^^67^. También es importante tener en cuenta las variantes anatómicas en el clivus y la base de cráneo, aun cuando son poco frecuentes, ya que se pueden encontrar como hallazgos incidentales y se debe decidir si se refiere, trata, observa o solo se informa. En el clivus se menciona el *canalis basilaris medianus*, sobre el cual en la literatura no alcanza un consenso respecto de su implicación clínica, ya que para algunos autores no la tiene y para otros puede ser una vía para facilitar la meningitis, pues las variantes anatómicas pueden favorecer que infecciones de origen nasofaríngeo lleguen a esta área ^68^^,^^69^.

Los sistemas de navegación quirúrgica han aumentado su frecuencia de uso en procedimientos endoscópicos y en cirugía de base de cráneo en otorrinolaringología, así como en neurocirugía, en la que la TCHC ha aumentado su presencia por mostrar un grado de certeza equivalente a la TCM y, en ocasiones, superior. Una precisión geométrica equivalente permite estudios preoperatorios para utilizarse en sistemas de navegación y dar seguimiento a sus instrumentos, de la patología a tratar y puntos de referencia anatómica ^18^^,^^59^^,^^70^^,^^71^.

La TCHC maxilofacial ha demostrado utilidad clínica en la evaluación de la colocación de un neuroestimulador en la fosa pterigopalatina como tratamiento de la migraña en racimo crónica. Se realiza una TCHC maxilofacial preoperatoria para evaluar anatomía de la fosa, la susceptibilidad de recibir el neurotransmisor y su tamaño. Se realiza una TCHC intraoperatoria para colocar el neurotransmisor y se compara con el estudio de TCHC maxilofacial postoperatorio para evaluar su correcta colocación, ya que puede invadir el seno maxilar o quedar fuera del área de estimulación del ganglio esfenopalatino. La TCHC intraoperatoria proporciona suficiente información para su colocación, pero la maxilofacial posoperatoria es superior para determinar su correcta colocación y, si se determina que está mal colocado, se puede reposicionar durante su recuperación sin requerir una hospitalización. Además, permite al neurólogo establecer los parámetros correctos de neuroestimulación al valorar el volumen 3D obtenido del estudio, ya que conoce la ubicación espacial del electrodo del neuroestimulador ^72^^-^^74^.

## CONCLUSIONES

La TCHC de la estructura craneofacial ha incrementado su utilidad fuera de la región maxilofacial, principalmente en las especialidades médicas relacionadas con área de cabeza y cuello, debido a las diferentes ventajas que presenta sobre la TCM en sus diferentes modalidades, incluido el gran detalle y resolución la anatomía complicada de esta región, los protocolos de menor radiación y su mayor accesibilidad. Con el avance del conocimiento por parte de médicos especialistas, cada vez más se encuentran aplicaciones de la TCHC en esta área, y en algunas especialidades ya es un referente para la evaluación preoperatoria, la observación, el diagnóstico, el plan de tratamiento y la evolución posoperatoria.

## Fuente de financiamiento:

Autofinanciado. La presente investigación no ha recibido ayudas específicas provenientes de agencias del sector público, sector comercial o entidades sin ánimo de lucro

## References

[1] Mozzo P, Procacci C, Tacconi A, Martini PT, Andreis IA (1998). A new volumetric CT machine for dental imaging based on the cone-beam technique: preliminary results. Eur Radiol.

[2] Miracle AC, Mukherji SK (2009). Conebeam CT of the head and neck, part 2: clinical applications. AJNR Am J Neuroradiol.

[3] Cavalcanti MG (2012). Cone beam computed tomographic imaging: perspective, challenges, and the impact of near-trend future applications. J Craniofac Surg.

[4] Palomo L, Palomo JM (2009). Cone beam CT for diagnosis and treatment planning in trauma cases. Dent Clin North Am.

[5] Suzuki S, Katada Y, Takayanagi T, Sugawara H, Ishikawa T, Yamamoto Y (2019). Evaluation of three-dimensional iterative image reconstruction in C-arm-based interventional cone-beam CT: A phantom study in comparison with customary reconstruction technique. Medicine (Baltimore).

[6] Fior D, Vacirca F, Leni D, Pagni F, Ippolito D, Riva L (2019). Virtual guidance of percutaneous transthoracic needle biopsy with C-Arm cone-beam CT: Diagnostic accuracy, risk factors and effective radiation dose. Cardiovasc Intervent Radiol.

[7] Orth RC, Wallace MJ, Kuo MD, Radiology TACotSoI (2008). -arm cone-beam CT: general principles and technical considerations for use in interventional radiology. J Vasc Interv Radiol.

[8] O'Connell AM, Karellas A, Vedantham S, Kawakyu-O'Connor DT (2018). Newer technologies in breast cancer imaging: dedicated cone-beam breast computed tomography. Semin Ultrasound CT MR.

[9] Raj S, Irani FG, Tay KH, Tan BS (2013). C-arm Cone beam computed tomography A new tool in the interventional suite. Ann Acad Med Singapore.

[10] Edwards R, Altalibi M, Flores-Mir C (2013). The frequency and nature of incidental findings in cone-beam computed tomographic scans of the head and neck region: a systematic review. J Am Dent Assoc.

[11] Dief S, Veitz-Keenan A, Amintavakoli N, McGowan R (2019). A systematic review on incidental findings in cone beam computed tomography (CBCT) scans. Dentomaxillofac Radiol.

[12] Monsarrat P, Galibourg A, Nasr K, Telmon N, Maret D (2019). Incidental findings in dental radiology are concerning for family doctors. Open Med (Wars).

[13] Carter L, Farman AG, Geist J, Scarfe WC, Angelopoulos C, Nair MK (2008). American Academy of Oral and Maxillofacial Radiology executive opinion statement on performing and interpreting diagnostic cone beam computed tomography. Oral Surg Oral Med Oral Pathol Oral Radiol Endod.

[14] Horner K, Islam M, Flygare L, Tsiklakis K, Whaites E. (2009). Basic principles for use of dental cone beam computed tomography: consensus guidelines of the European Academy of Dental and Maxillofacial Radiology. Dentomaxillofac Radiol.

[15] Edwards R, Alsufyani N, Heo G, Flores-Mir C (2014). The frequency and nature of incidental findings in large-field cone beam computed tomography scans of an orthodontic sample. Prog Orthod.

[16] Vos WD, Casselman J, Swennen G (2009). Cone-beam computerized tomography (CBCT) imaging of the oral and maxillofacial region: a systematic review of the literature.

[17] Lechuga L, Weidlich GA (2016). Cone Beam CT vs. Fan Beam CT: A comparison of image quality and dose delivered between two differing CT imaging modalities. Cureus.

[18] Watanabe H, Honda E, Tetsumura A, Kurabayashi T (2011). A comparative study for spatial resolution and subjective image characteristics of a multi-slice CT and a cone-beam CT for dental use. Eur J Radiol.

[19] Brullmann D, Schulze RK (2015). Spatial resolution in CBCT machines for dental/maxillofacial applications-what do we know today?. Dentomaxillofac Radiol.

[20] Barghan S, Tahmasbi Arashlow M, Nair MK (2016). Incidental findings on cone beam computed tomography studies outside of the maxillofacial skeleton. Int J Dent.

[21] Ludlow JB, Timothy R, Walker C, Hunter R, Benavides E, Samuelson DB (2015). Effective dose of dental CBCT-a meta analysis of published data and additional data for nine CBCT units. Dentomaxillofac Radiol.

[22] Nardi C, Salerno S, Molteni R, Occhipinti M, Grazzini G, Norberti N (2018). Radiation dose in non-dental cone beam CT applications: a systematic review. Radiol Med.

[23] Brenner DJ, Hall EJ (2007). Computed tomography--an increasing source of radiation exposure. N Engl J Med.

[24] Braganza MZ, Kitahara CM, Berrington de González A, Inskip PD, Johnson KJ, Rajaraman P (2012). Ionizing radiation and the risk of brain and central nervous system tumors: a systematic review. Neuro Oncol.

[25] Meulepas JM, Ronckers CM, Smets A, Nievelstein RAJ, Gradowska P, Lee C (2019). Radiation exposure from pediatric CT scans and subsequent cancer risk in the Netherlands. J Natl Cancer Inst.

[26] Lee CY, Koval TM, Suzuki JB (2015). Low-dose radiation risks of computerized tomography and cone beam computerized tomography: Reducing the fear and controversy. J Oral Implantol.

[27] Davis F, Il'yasova D, Rankin K, McCarthy B, Bigner DD (2011). Medical diagnostic radiation exposures and risk of gliomas. Radiat Res.

[28] Loubele M, Bogaerts R, Van Dijck E, Pauwels R, Vanheusden S, Suetens P (2009). Comparison between effective radiation dose of CBCT and MSCT scanners for dentomaxillofacial applications. Eur J Radiol.

[29] Hofmann E, Schmid M, Sedlmair M, Banckwitz R, Hirschfelder U, Lell M (2014). Comparative study of image quality and radiation dose of cone beam and low-dose multislice computed tomography--an in-vitro investigation. Clin Oral Investig.

[30] Nardi C, Talamonti C, Pallotta S, Saletti P, Calistri L, Cordopatri C (2017). Head and neck effective dose and quantitative assessment of image quality: a study to compare cone beam CT and multislice spiral CT. Dentomaxillofac Radiol.

[31] Almashraqi AA, Ahmed EA, Mohamed NS, Barngkgei IH, Elsherbini NA, Halboub ES (2017). Evaluation of different low-dose multidetector CT and cone beam CT protocols in maxillary sinus imaging: part I-an in vitro study. Dentomaxillofac Radiol.

[32] Nardi C, Borri C, Regini F, Calistri L, Castellani A, Lorini C (2015). Metal and motion artifacts by cone beam computed tomography (CBCT) in dental and maxillofacial study. Radiol Med.

[33] Fontolliet M, Bornstein MM, von Arx T (2019). Characteristics and dimensions of the infraorbital canal: a radiographic analysis using cone beam computed tomography (CBCT). Surg Radiol Anat.

[34] Sinanoglu A, Orhan K, Kursun S, Inceoglu B, Oztas B (2016). Evaluation of optic canal and surrounding structures using cone beam computed tomography: considerations for maxillofacial surgery. J Craniofac Surg.

[35] Papathanassiou S, Koch T, Suhling MC, Lenarz T, Durisin M, Stolle SRO

[36] Tschopp M, Bornstein MM, Sendi P, Jacobs R, Goldblum D (2014). Dacryocystography using cone beam CT in patients with lacrimal drainage system obstruction. Ophthalmic Plast Reconstr Surg.

[37] Berg BI, Dagassan-Berndt D, Goldblum D, Kunz C (2015). Cone-beam computed tomography for planning and assessing surgical outcomes of osteo-odonto-keratoprosthesis. Cornea.

[38] Norris JM, Kishikova L, Avadhanam VS, Koumellis P, Francis IS, Liu CS (2015). Comparison of 640-Slice Multidetector Computed Tomography Versus 32-Slice MDCT for Imaging of the Osteo-odonto-keratoprosthesis Lamina. Cornea.

[39] Qiao X, Liu W, Cao Y, Miao C, Yang W, Su N (2018). Performance of different imaging techniques in the diagnosis of head and neck cancer mandibular invasion: A systematic review and meta-analysis. Oral Oncol.

[40] Sarrión Pérez MG, Bagán JV, Jiménez Y, Margaix M, Marzal C (2015). Utility of imaging techniques in the diagnosis of oral cancer. J Craniomaxillofac Surg.

[41] Hakim SG, Wieker H, Trenkle T, Sieg P, Konitzer J, Holl-Ulrich K (2014). Imaging of mandible invasion by oral squamous cell carcinoma using computed tomography, cone-beam computed tomography and bone scintigraphy with SPECT. Clin Oral Investig.

[42] Linz C, Müller-Richter UD, Buck AK, Mottok A, Ritter C, Schneider P (2015). Performance of cone beam computed tomography in comparison to conventional imaging techniques for the detection of bone invasion in oral cancer. Int J Oral Maxillofac Surg.

[43] Ruivo J, Mermuys K, Bacher K, Kuhweide R, Offeciers E, Casselman JW (2009). Cone beam computed tomography, a low-dose imaging technique in the postoperative assessment of cochlear implantation. Otol Neurotol.

[44] Hodez C, Griffaton-Taillandier C, Bensimon I (2011). Cone-beam imaging: applications in ENT. Eur Ann Otorhinolaryngol Head Neck Dis.

[45] Cakli H, Cingi C, Ay Y, Oghan F, Ozer T, Kaya E (2012). Use of cone beam computed tomography in otolaryngologic treatments. Eur Arch Otorhinolaryngol.

[46] Zojaji R, Naghibzadeh M, Mazloum Farsi Baf M, Nekooei S, Bataghva B, Noorbakhsh S (2015). Diagnostic accuracy of cone-beam computed tomography in the evaluation of chronic rhinosinusitis. ORL J Otorhinolaryngol Relat Spec.

[47] Roman RA, HedeSIu M, Gersak M, Fidan F, BACiuT G, BACiuT M (2016). Assessing the prevalence of paranasal sinuses anatomical variants in patients with sinusitis using Cone Beam Computer Tomography. Clujul Med.

[48] Guldner C, Ningo A, Voigt J, Diogo I, Heinrichs J, Weber R (2013). Potential of dosage reduction in cone-beam-computed tomography (CBCT) for radiological diagnostics of the paranasal sinuses. Eur Arch Otorhinolaryngol.

[49] Shokri A, Miresmaeili A, Farhadian N, Falah-Kooshki S, Amini P, Mollaie N (2017). Effect of changing the head position on accuracy of transverse measurements of the maxillofacial region made on cone beam computed tomography and conventional posterior-anterior cephalograms. Dentomaxillofac Radiol.

[50] Khojastepour L, Mirhadi S, Mesbahi SA (2015). Anatomical variations of ostiomeatal complex in CBCT of patients seeking rhinoplasty. J Dent (Shiraz).

[51] Bance M, Zarowski A, Adamson RA, Casselman JW (2018). New imaging modalities in otology. Adv Otorhinolaryngol.

[52] Debeaupte M, Hermann R, Pialat JB, Martinon A, Truy E, Ltaief Boudrigua A (2019). Cone beam versus multi-detector computed tomography for detecting hearing loss. Eur Arch Otorhinolaryngol.

[53] Stutzki M, Jahns E, Mandapathil MM, Diogo I, Werner JA, Guldner C (2015). Indications of cone beam CT in head and neck imaging. Acta Otolaryngol.

[54] Hiraumi H, Suzuki R, Yamamoto N, Sakamoto T, Ito J (2016). The sensitivity and accuracy of a cone beam CT in detecting the chorda tympani. Eur Arch Otorhinolaryngol.

[55] Zhang Z, Yin H, Wang Z, Li J, Lv H, Zhao P, Yang Z, Wang Z (2019). Imaging re-evaluation of the tympanic segment of the facial nerve canal using cone-beam computed tomography compared with multi-slice computed tomography. Eur Arch Otorhinolaryngol.

[56] immerman JN, Lee J, Pliska BT (2017). Reliability of upper pharyngeal airway assessment using dental CBCT: a systematic review. Eur J Orthod.

[57] Chen H, Aarab G, Parsa A, de Lange J, van der Stelt PF, Lobbezoo F (2016). Reliability of three-dimensional measurements of the upper airway on cone beam computed tomography images. Oral Surg Oral Med Oral Pathol Oral Radiol.

[58] Chen H, van Eijnatten M, Aarab G, Forouzanfar T, de Lange J, van der Stelt P (2018). Accuracy of MDCT and CBCT in three-dimensional evaluation of the oropharynx morphology. Eur J Orthod.

[59] Xu J, Reh DD, Carey JP, Mahesh M, Siewerdsen JH (2012). Technical assessment of a cone-beam CT scanner for otolaryngology imaging: image quality, dose, and technique protocols. Med Phys.

[60] Vandevenne JE, Peuskens D, Wijnen L, Wuyts J (2016). Cone-beam CT to assess bony fusion following anterior cervical interbody fusion. Eur Spine J.

[61] Razi T, Niknami M, Alavi Ghazani F (2014). Relationship between Hounsfield Unit in CT Scan and Gray Scale in CBCT. J Dent Res Dent Clin Dent Prospects.

[62] Bastami F, Shahab S, Parsa A, Abbas FM, Noori Kooshki MH, Namdari M (2018). Can gray values derived from CT and cone beam CT estimate new bone formation? An in vivo study. Oral Maxillofac Surg.

[63] Razi T, Emamverdizadeh P, Nilavar N, Razi S (2019). Comparison of the Hounsfield unit in CT scan with the gray level in cone-beam CT. J Dent Res Dent Clin Dent Prospects.

[64] Mladina R, Antunovic R, Cingi C, Muluk NB, Skitarelic N, Malic M (2017). An anatomical study of pneumatized crista galli. Neurosurg Rev.

[65] Akkoca Kaplan F, Bayrakdar IS, Bilgir E (2020). Incidence of anomalous canals in the base of the skull: a retrospective radio-anatomical study using cone-beam computed tomography. Surg Radiol Anat.

[66] Policeni BA, Smoker WR (2015). Imaging of the skull base: anatomy and pathology. Radiol Clin North Am.

[67] Quirk B, Connor S (2020). Skull base imaging, anatomy, pathology and protocols. Pract Neurol.

[68] Bayrak S, Goller Bulut D, Orhan K (2019). Prevalence of anatomical variants in the clivus: fossa navicularis magna, canalis basilaris medianus, and craniopharyngeal canal. Surg Radiol Anat.

[69] Syed AZ, Zahedpasha S, Rathore SA, Mupparapu M (2016). Evaluation of canalis basilaris medianus using cone-beam computed tomography. Imaging Sci Dent.

[70] von Wilmowsky C, Bergauer B, Nkenke E, Neukam FW, Neuhuber W, Lell M (2015). A new, highly precise measurement technology for the in vitro evaluation of the accuracy of digital imaging data. J Craniomaxillofac Surg.

[71] Talks BJ, Jolly K, Burton H, Koria H, Ahmed SK (2019). Cone-beam computed tomography allows accurate registration to surgical navigation systems: A multidevice phantom study. Am J Rhinol Allergy.

[72] Assaf AT, Klatt JC, Blessmann M, Kohlmeier C, Friedrich RE, Pohlenz P (2015). Value of intra- and post-operative cone beam computed tomography (CBCT) for positioning control of a sphenopalatine ganglion neurostimulator in patients with chronic cluster headache. J Craniomaxillofac Surg.

[73] Jurgens TP, Schoenen J, Rostgaard J, Hillerup S, Lainez MJ, Assaf AT (2014). Stimulation of the sphenopalatine ganglion in intractable cluster headache: expert consensus on patient selection and standards of care. Cephalalgia.

[74] Narouze SN (2010). Role of sphenopalatine ganglion neuroablation in the management of cluster headache. Curr Pain Headache Rep.

